# Detection and Prediction of the Early Thermal Runaway and Control of the Li-Ion Battery by the Embedded Temperature Sensor Array

**DOI:** 10.3390/s23115049

**Published:** 2023-05-25

**Authors:** Hengyi Zhang, Xiaoshan Zhang, Wenwu Wang, Ping Yu

**Affiliations:** College of Material Science and Engineering, Sichuan University, Chengdu 610064, China; enactus_zhy@163.com (H.Z.); zhills@hotmail.com (X.Z.); www1492@163.com (W.W.)

**Keywords:** Li-ion battery (LIB), temperature sensor array, positive temperature coefficient resistor (PTCR), BaTiO_3_ ceramics

## Abstract

Sorts of Li-ion batteries (LIB) have been becoming important energy supply and storage devices. As a long-standing obstacle, safety issues are limiting the large-scale adoption of high-energy–density batteries. Strategies covering materials, cell, and package processing have been paid much attention to. Here, we report a flexible sensor array with fast and reversible temperature switching that can be incorporated inside batteries to prevent thermal runaway. This flexible sensor array consists of PTCR ceramic sensors combined with printed PI sheets for electrodes and circuits. Compared to room temperature, the resistance of the sensors soars nonlinearly by more than three orders of magnitude at around 67 °C with a 1 °C/s rate. This temperature aligns with the decomposition temperature of SEI. Subsequently, the resistance returns to normal at room temperature, demonstrating a negative thermal hysteresis effect. This characteristic proves advantageous for the battery, as it enables a lower-temperature restart after an initial warming phase. The batteries with an embedded sensor array could resume their normal function without performance compromise or detrimental thermal runaway.

## 1. Introduction

Various Li-ion batteries (LIBs) have been becoming the predominant energy supply and storage devices [1,2,3]. The safety issues of LIB represented by thermal runaway (TR) are still not completely avoidable in many practical applications at the current stage. Thermal abuse, mechanical abuse, and electrical abuse [4,5] are regarded as the most abusive conditions that induce LIB to deviate from a healthy working state and finally lead to TR [5]. Sorts of strategies have been considered to reduce the possibility of TR occurrence, covering the materials level, cell level, and package level systematically [1,6,7]. Considerable research efforts have been dedicated to exploring advanced materials for various components of LIBs. These include the modification of electrode microstructures and material compositions [5,6,8], enhancing the electrolyte’s vaporization point and fire retardancy [5,8], developing temperature-responsive and shutdown separators [5,9,10,11,12], and other related areas.

Since the TR process is accompanied by changes in many physical and chemical signals, such as voltage, current, temperature, pressure, and gas [13], the safety of the battery can be inferred by using different sensors to judge them. In the past decades, studies on the TR of LIB have promoted various methods to detect [14,15,16,17] the internal temperature of LIB. Enlightened by these works, the technologies of monitoring and controlling the state of charge (SOC) and the state of health (SOH) [6] by physical sensors and virtual sensors while LIB is working have been proposed [18,19]. Among them, two kinds of positive temperature coefficient resistance (PTCR) components have been incorporated into LIB. The first type involves the incorporation of PTC ceramic rings within the cap structure of commercial cylindrical LIBs. The second type comprises various special composite polymer materials that exhibit the PTCR effect. These materials have been extensively researched and designed as potential solutions to effectively mitigate the occurrence of TR in LIBs [1,5]. The PTCR effect is characterized by a significant nonlinear increase in resistance [20] at a certain temperature point or within a very narrow temperature range as the temperature increases.

For cap-design cylindrical LIBs, the PTC thermistor works as a conductive component to protect the single cell from excessive current because its resistance increases with temperature. However, for those batteries designed for high-discharge-rate applications as high as 20 A, PTC thermistors are not accepted [1]. Furthermore, PTC thermistors are not found in commercial prismatic or pouch batteries. For the composite polymers, the PTCR effect stems from the glass transformation of the polymer matrix. This transformation leads to a volume expansion of the polymer matrix, causing the conductive path of conductive particles (acting as filler) to be disrupted [7,20]. It is expected that the resistance of the composite polymer coated on the cathode will rise rapidly to turn off the LIB before the early stage of thermal runaway. However, the strategy of LIB protection by the composite polymer materials’ PTCR effects is limited in practical use. Firstly, most of the polymer materials show the PTCR effect at a temperature higher than 90 °C [21,22,23,24,25,26,27,28]. Notably, the reported temperature of the SEI layer (solid-electrolyte interphase) decomposing (around 70 °C) or breakdown would be 90–100 °C [29], which is related to the first stage of the thermal runaway reaction. Secondly, the existing PTCR composite polymer is coated on the cathode, which is not reliable for reacting quickly in other places. For example, it could be more than 20 °C lower than that of the core part (>160 °C) [30] under a thermal abuse condition. As a result, the LIB could not be protected effectively. Thirdly, the room-temperature electrical resistivity of the composite polymer would increase with the heating/cooling cycles [31]. It indicates that the composite polymer materials may cause a worsening of LIB service characteristics by increasing cell resistance. Meanwhile, the intrinsic thermal degradation of the polymer [32] makes it hard for the composite polymer to ensure the stability of the PTCR effect for a long time.

BaTiO_3_-based ceramics with PTCR effects have been widely used as temperature sensors, circuit-limiting components, switches, and thermal fuses [20,33]. The resistance of the BaTiO_3_-based ceramics can rise by several magnitudes around the Curie Temperature (*T*_c_). The *T*_c_ could be modulated over a wide temperature range by doping chemical elements. Specifically, the BaTiO_3_-based ceramics show satisfactory electric stability and reproducibility in practical application. In this work, BaTiO_3_-based ceramics with the PTCR effect were investigated as the embedded temperature sensor for pouch LIB batteries to achieve multi-point temperature detection. The BaTiO_3_-based PTCR ceramic sensor was prepared by the tape casting process. A flexible temperature sensor array was fabricated with the polyimide (PI) sheet and the hot-pressing process. The temperature sensor array was then packed into a pouch cell. The *T*_c_ of the embedded sensors is modulated from 67, 70, 80, and 90 °C, respectively, to satisfy different switch temperatures. The temperature response of the sensors was measured and reported.

## 2. Materials and Methods

### 2.1. Ceramics Preparation

0.99(Ba_1-x_Sr_x_Zr_y_Y_0.005_Ti_0.995-y_O_3_)-0.01(Bi_0.5_Na_0.5_TiO_3_) (BSZYT-BNT) ceramics were prepared as follows: Firstly, the BSZYT-BNT precursor gel was prepared by the sol-gel method. Barium acetate, strontium acetate, sodium acetate trihydrate, zirconium nitrate pentahydrate, yttrium nitrate hexahydrate, and bismuth nitrate pentahydrate were used as raw materials. Deionized water, acetic acid, ethanol, and ethylene glycol methyl ether were used as solvents. Secondly, the BSZYT-BNT powder was produced by calcining the dry precursor gel at 750 °C and then grinding. Thirdly, the BSZYT-BNT ceramics were formed by the tape casting process and sintered at 1270 °C, and then the ceramic slices sized at 2.5 mm × 2.5 mm were obtained by cutting processing. The thickness of the prepared ceramic slices was ~115 µm. Au electrodes were sputtered on both sides of the ceramic slices.

### 2.2. Sensor Preparation

The prepared BSZYT-BNT ceramic slices prepared by the tape casting process serve as the temperature sensor of the temperature sensor array, which is placed between two layers of PI sheets printed with copper electrodes and circuits. Then the double PI sheets were joined together by hot-pressing processing. The thickness of the PI sheet is 30 µm. The sensor array was designed as a sandwich structure. The sensor elements are located between the two PI sheets. The copper electrodes were fabricated on one of the sides of the PI sheets, which is the inner side of the two PI sheets. All the copper electrodes and the sensor elements were sealed inside the sensor array.

### 2.3. Measurement and Characterization

The DC resistance of each prepared BSZYT-BNT ceramic sensor was measured by an electrometer (Keithley Model 6517B) with an operating voltage of 2 V in the test box with a programmable temperature controller. The resistance-temperature performance of the prepared ceramic sensor was obtained in a continuous heating and cooling environment. After the prepared sensor array was embedded in the battery, the resistance-temperature response of the sensor array was tested in two different scenarios. The first scenario is that the battery was not charging or discharging, called the static measurement. The second scenario is that the battery was charging and discharging, respectively, which is called the dynamic measurement. For the static measurement, the internal ambient temperature of the testing box varied from room temperature to 100 °C with a 5 °C/min changing rate and was held at 100 °C for 60 min before cooling down naturally. For the dynamic measurement, the temperature of the testing box was kept constant at 55 °C and 60 °C, respectively, while the battery was charging and discharging.

## 3. Results and Discussion

### 3.1. PTCR Properties

#### 3.1.1. Ceramics

**Figure 1:**
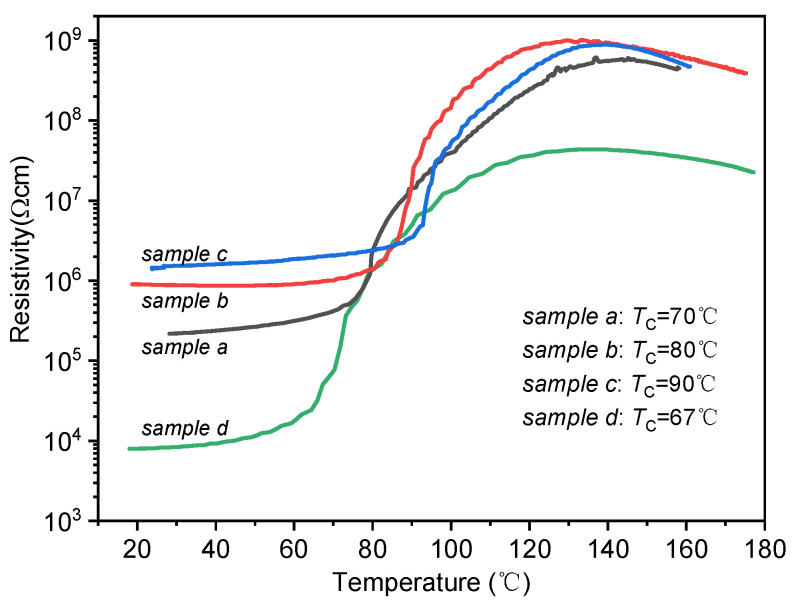
The prepared BSZYT-BNT ceramics show different *T*_c_ by changing the Sr and Zr stoichiometric ratios.

According to the analysis of the SEI layer on graphite negative electrodes in lithium-ion batteries, the first stage of the thermal runaway reaction is the breakdown of the thin SEI layer. This breakdown typically occurs at around 90 °C [34,35,36,37]. Furthermore, the SEI layer may decompose at a relatively lower temperature of 69 °C [38]. Therefore, detecting the change in the SEI layer at an early stage is important. In the present work, the BSZYT-BNT ceramics with different *T*_c_ were prepared by adjusting the proportions of Sr and Zr. The *T*_c_ of the prepared BSZYT-BNT ceramics is 67 °C, 70 °C, 80 °C, and 90 °C, respectively (see Figure 1).

**Figure 2:**
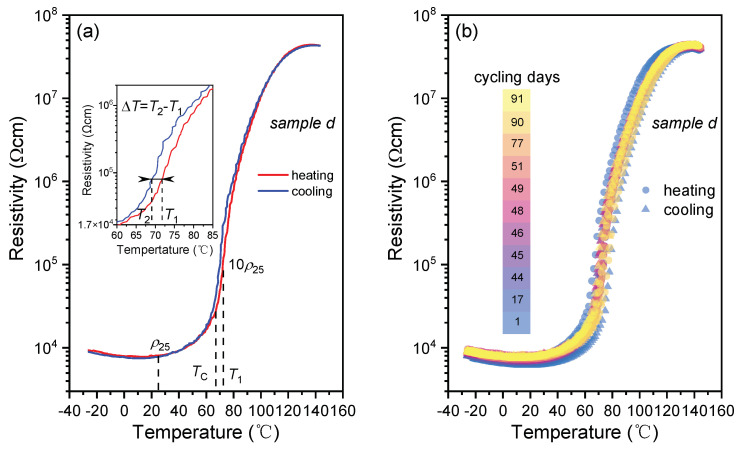
The PTCR characteristics of the prepared BSZYT-BNT ceramics. (**a**) The resistivity temperature response of sample *d* in Figure 1 is an example. (**b**) The reproducibility of *ρ*-*T* curves for sample *d*.

PTCR characteristics of the prepared BSZYT-BNT ceramics are evaluated by the following parameters: the room temperature electric resistivity (*ρ*_25_), the *T*_c_ values, the PTCR jump (calculated by log(*ρ*_max_/*ρ*_min_)), and the thermal hysteresis Δ*T* (temperature difference between the temperatures of *T*_2_ and *T*_1_, *T*_2_ and *T*_1_ corresponding to the 10*ρ*_25_ values of the BSZYT-BNT ceramics while cooling and heating, respectively, see Figure 2a).

Figure 2a shows the resistivity temperature response of sample *d* in Figure 1. The room temperature resistivity (*ρ*_25_) of sample *d* is 8.1 × 10^3^ Ωcm. In the first stage, the resistivity rises mildly from 27 °C to 62 °C. An appreciable increase in electrical resistivity occurs from 62 °C to 65 °C. After that, a more dramatic change starts from 65 °C to 80 °C, when the electrical resistivity increases from 2.3 × 10^4^ Ωcm to 7.9 × 10^5^ Ωcm and then reaches its maximum value of 4.4 × 10^7^ Ωcm at 137 °C. An almost plateau is observed in the temperature range from 110 °C to 140 °C. The *T*_c_ value is 67 °C, which could match the SEI decomposing temperature [38] and the self-heating temperature of practical commercial LIB [29]. The PTCR jump reaches 3.75 and the thermal hysteresis Δ*T* is around −3.3 °C resulting from the inevitable thermal hysteresis in the phase transition of BaTiO_3_. In a practical case, the negative value of Δ*T* is profit for the safety of the battery, which means that the battery could be restarted at a lower temperature after early warning.

Figure 2b shows the reproducibility of the resistivity-temperature response curves of the sample in Figure 2a. After continuous testing for more than 90 days, the results show that the *T*_c_ and the PTCR jump keep values of 67 °C and 3.75 °C stably. The ratio of resistivity change at room temperature is initially less than 20% within the first 14 days and then stabilizes over time. It suggests that the PTCR characteristics of the sample show good stability, which is a prerequisite for temperature-control switch applications in LIB. All the PTCR performances of the prepared samples in Figure 1 are listed in Table 1. The results suggest that the prepared BSZYT-BNT ceramics could be expected to be an effective temperature switch sensor to protect the battery before the occurrence of the exothermic side reaction.

#### 3.1.2. Temperature Sensor Array

**Figure 3:**
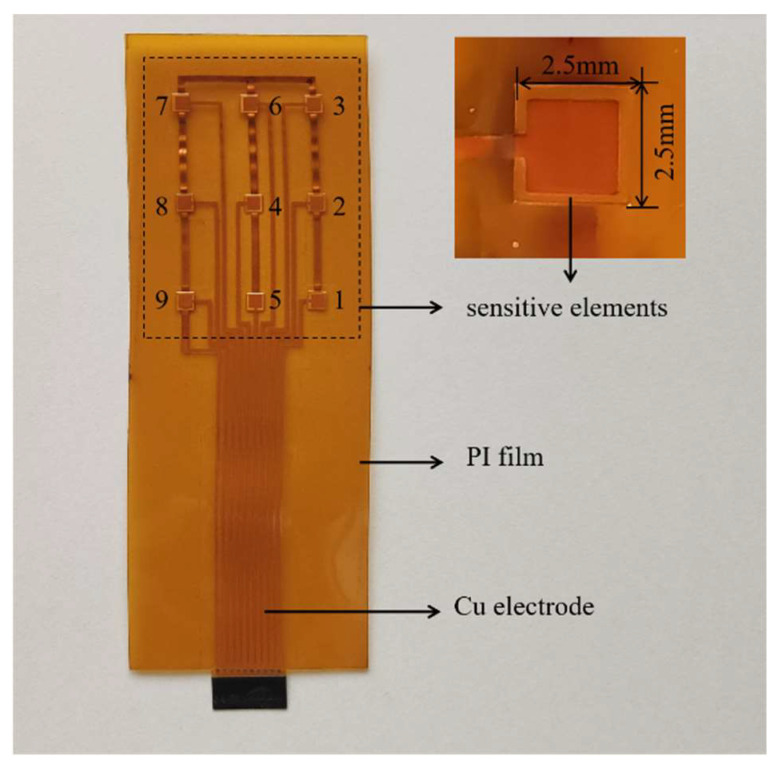
A photo of the temperature sensor array, where each sensor element is numbered from 1 to 9, and an enlarged view of one typical sensor element on the right side.

**Figure 4:**
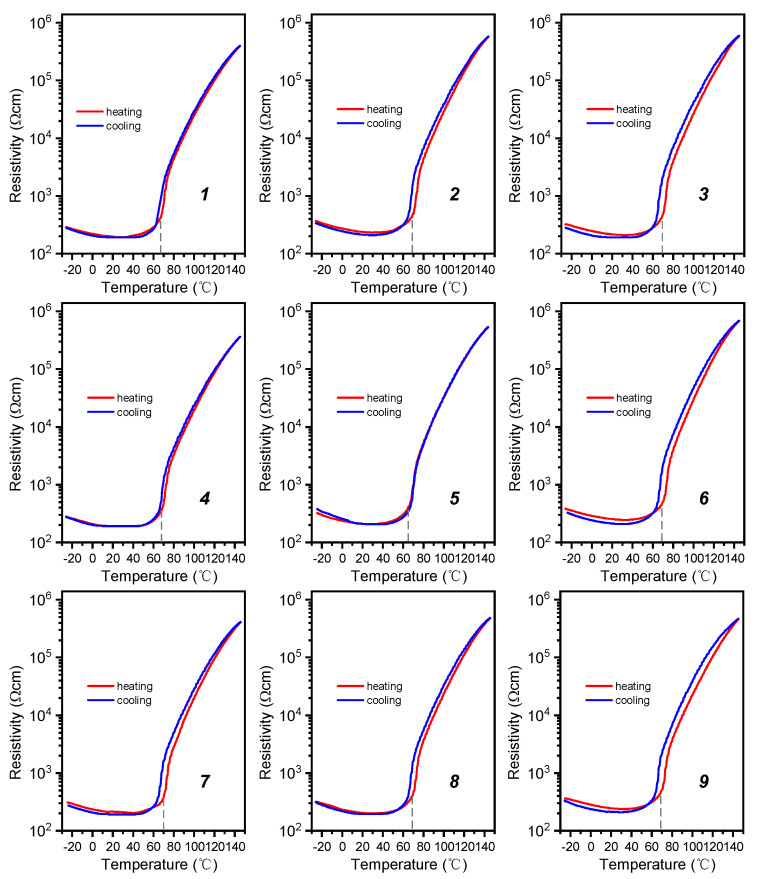
*ρ*-*T* curves for each sensitive element of a temperature sensor array and *T*_c_ of the heating section are noted by gray dashed lines. The number ranged 1–9 in each subgraph corresponds to the position of each sensitive element as marked in Figure 3.

A thin and flexible BSZYT-BNT ceramic sensor array containing nine sensors was fabricated by using PI sheets printed with copper electrodes and circuits as covers. A photograph of the prepared sensor array is shown in Figure 3. It is designable for the fabrication of the temperature sensor array by adjusting the position and number of the sensitive elements, as well as the size of the PI sheets. Therefore, various LIBs with different sizes and shapes can be matched. The *T*_c_ of the sensors in the prepared sensor array is around 67 °C. Before embedding the battery, the PTCR performance of each sensor was tested; the *ρ*-*T* curves are depicted in Figure 4. Highly similar PTCR effects are performed by the nine sensitive elements, indicating good consistency in multi-point temperature detection. Compared with the measurement results in Figure 2a, it suggests that the encapsulation of PI films does not have any negative impacts on the temperature response of the sensors.

**Figure 5:**
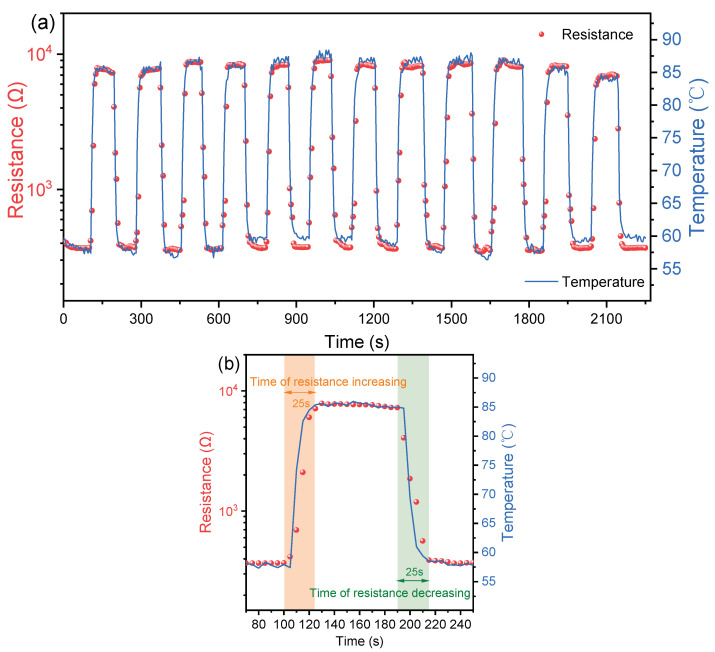
(**a**) The temperature shock test ranged from 60 °C to 85 °C for the prepared sensor array for 12 cycles. (**b**) The details of the first cycle.

The temperature shock test from 60 °C to 85 °C was carried out on the prepared sensor array for 12 cycles to evaluate the temperature response time. The values of resistance and temperature were recorded synchronously while the heat shock and cool shock were carried out, respectively. The results of one of the sensors in the prepared sensor array are shown in Figure 5a. The detailed data is given in Figure 5b.

Figure 5b gives a clear observation of the resistance response time of the sensor in the sensor array while a heating and cooling shock were carried out, respectively. It indicates that it takes around 25 s for the sensor to finish the resistance rising from 0.39 kΩ to 8 kΩ while the sensor array was moved into the 85 °C oil bath from a 60 °C oil bath in a thermal equilibrium state. It suggests that the temperature response of the sensor, ranging from 60 °C to 85 °C, is around 1 °C/s. The reported temperature rise rates in the early TR process are listed in Table 2. Although there is inconsistency among those results due to different types of batteries and SOC, it is generally demonstrated that the temperature rise rate is lower than 1 °C/s in the temperature range of 60–85 °C. During the cooling, the sensor showed a similar response time of 25 s as well. Despite the sensor being covered by PI films, a fast temperature response of around 1 °C/s could be achieved by the prepared sensor array.

### 3.2. Static Measurement Inside the Battery

**Figure 6:**
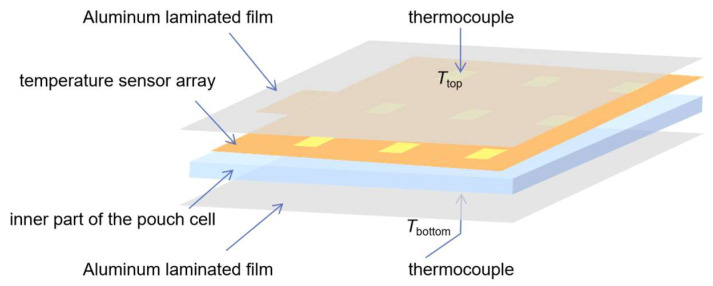
Structure schematic diagram of the pouch cell embedded with the sensor array.

**Figure 7:**
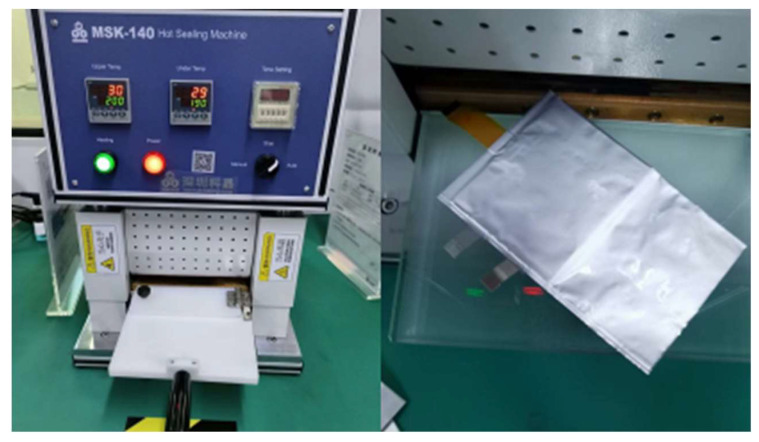
The optical photograph of the fabrication of the pouch cell embedded with the sensor array.

**Figure 8:**
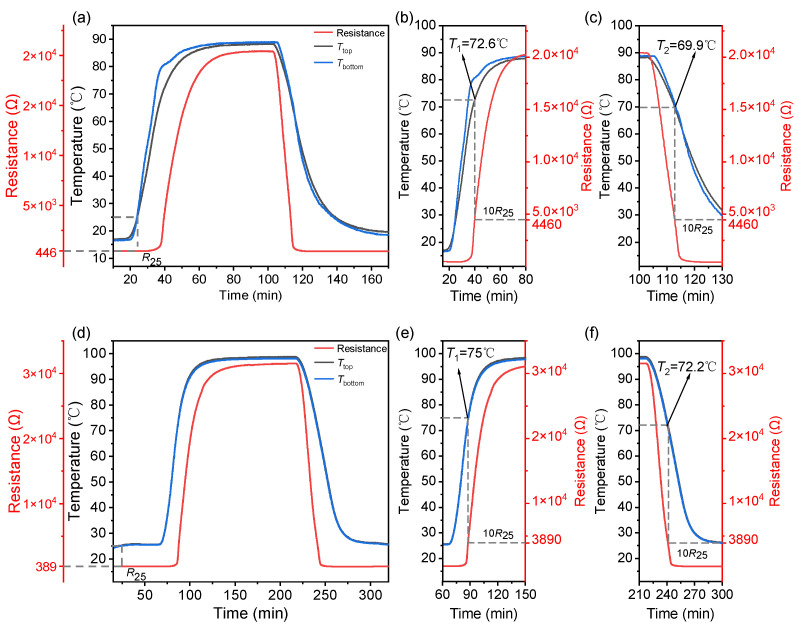
Temperature and resistance result in the static measurements conducted at the maximum temperature at (**a**) 90 °C and (**d**) 100 °C. The enlarged views around 70 °C during heating and cooling parts are shown in (**b**,**e**) and (**c**,**f**), respectively.

The prepared sensor array was inserted into a pouch cell, as shown in Figure 6. The implantation of the sensor array is compatible with the pouch cell assembly process (see Figure 7). Compared to the PTC ring in cylindrical LIBs or the PTC cathode, this incorporation process does not require additional design for LIB. The resistance temperature response of the sensor array was tested while the battery was in a static state, which means it was not in a charge or discharge state. The battery embedded with a sensor array was placed in the test box with a programmable temperature controller, which was heated from room temperature to a certain temperature of 5 °C/min and then cooled down naturally. The tests were conducted twice for the highest preset temperatures of 90 °C and 100 °C, respectively, and are noted as test 1 (see Figure 8a–c) and test 2 (see Figure 8d–f). Two K-type thermocouples were laid on the top and bottom surfaces of the battery to record the surface temperatures, which are noted as *T*_top_ and *T*_bottom_ (see Figure 6).

As shown in Figure 8, in both tests, the resistance of the sensor is almost unchanged around *R*_25_ in each test below the temperature of 50 °C. This temperature corresponds to the top side of the battery because the array was near the top side (see Figure 6) of the battery. In test 1, as shown in Figure 8b, an obvious jump in resistance is performed at 65 °C, and then it rises to 10 times *R*_25_, i.e., 4460 Ω at 72.6 °C. Around 90 °C, the resistance reaches 2.0 × 10^4^ Ω. During the following cooling section, the resistance drops rapidly to 4460 Ω at 69.9 °C, as marked in Figure 8c. In test 2, a larger PTCR jump is observed. As is shown in Figure 8d,e, *R*_25_ is 389 Ω, and the resistance soars to 3890 from 65 °C to 75 °C, then rises to 1.8 × 10^4^ Ω at 90 °C and 3.1 × 10^4^ Ω at 100 °C, which is almost two orders of magnitude of *R*_25_. After a holding stage of temperature at 100 °C for 60 min, the battery naturally cooled down. The resistance value of the sensor decreases as the ambient temperature cools. A clear observation is shown in Figure 8f. It reveals that the resistance of the sensor decreases to 3890 Ω at 72.2 °C. The thermal hysteresis Δ*T* in test 1 and test 2 is −2.7 °C and −2.8 °C, respectively, indicating similar switching behavior. The static measurements confirm the repeatability in temperature detection of the temperature sensor array in the internal environment of LIB.

### 3.3. Dynamic Measurement Inside the Battery

**Figure 9:**
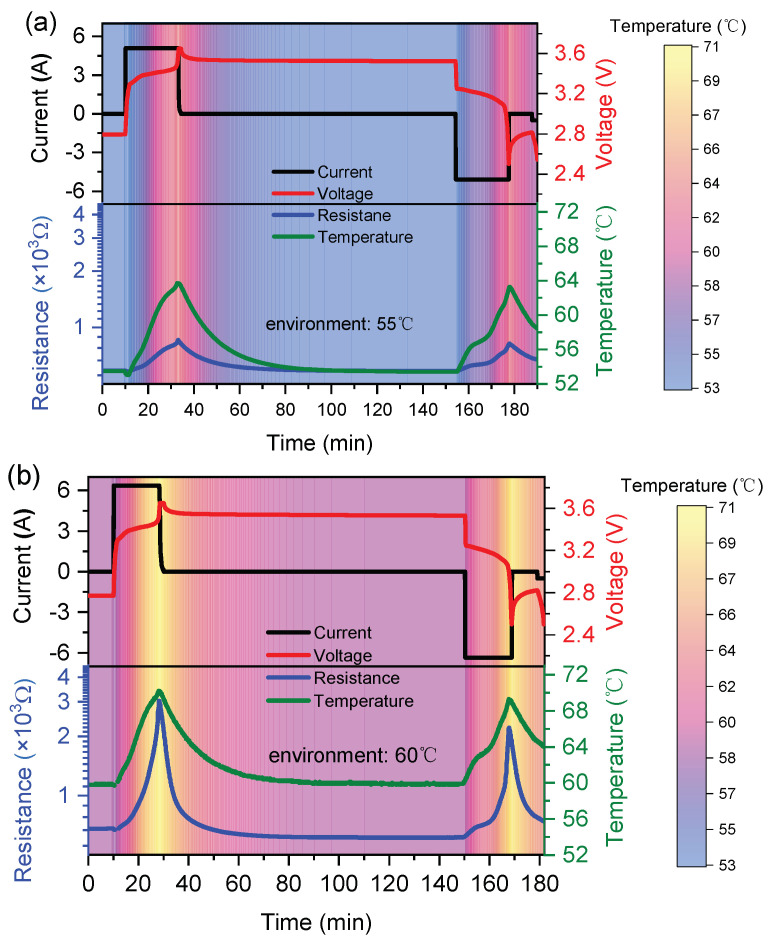
Current and voltage changes of the LIB during the charging and discharging processes, and the corresponding resistance changes of the embedded PTCR thermistor in (**a**) 55 °C and (**b**) 60 °C external thermostat environments, respectively.

The dynamic test of the sensor array was carried out while the battery was charging and discharging. The charging and discharging behavior of the battery and the resistance change of the sensors in the array are recorded simultaneously. The results are shown in Figure 9. In order to detect the resistance change of the sensor near the *T*_c_ region, the tests were conducted at 2C and 2.5C rates at ambient temperatures of 55 °C and 60 °C, respectively. The battery embedded with a sensor array was kept in the test box at 55 °C and 60 °C for 60 min so that the battery could obtain a total thermal balance with the ambient temperature before the test. The test starts with a 2C rate charge at 55 °C and a 2.5C rate at 60 °C, respectively. The subsequent discharging operations were not started until the internal temperature of the battery cooled down to 55 °C or 60 °C completely. Then, the test restarts with a 2C rate discharge at 55 °C and a 2.5C rate discharge at 60 °C, respectively. An NTCR (negative temperature coefficient resistance) thermistor was used in the sensor array to detect the internal temperature in real-time as a reference. The temperature line (green line) shown in Figure 9 is indicated by the NTCR thermistor. The resistances of the sensor at 25 °C, 55 °C, and 60 °C are 389 Ω (shown in Figure 8d), 610 Ω, and 720 Ω, respectively.

As shown in Figure 9, the internal temperature of the battery would rise while working. Compared to the charging stage, less heat is generated during the discharging stage. The related values are listed in Table 3.

**Figure 10:**
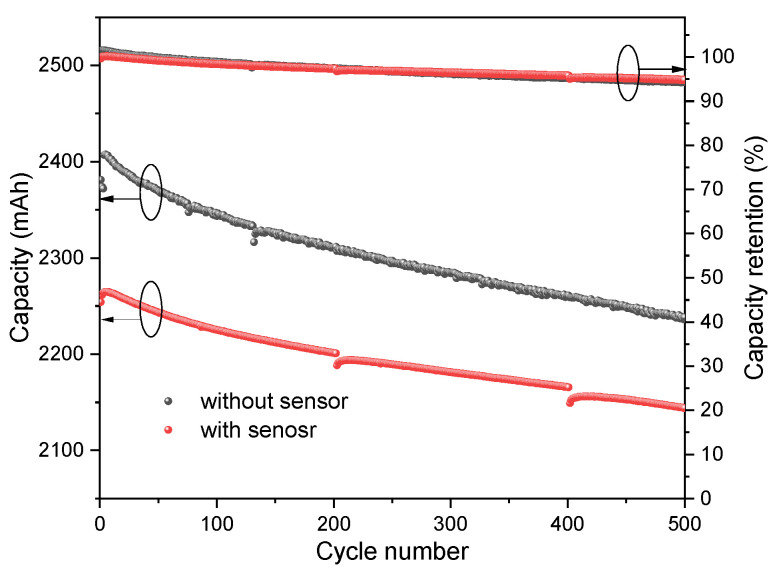
Cycling performance in charging and discharging the battery with/without the temperature sensor array buried at 55 °C and 1C.

For the 2C rate charging at the ambient temperature of 55 °C, the maximum internal temperature of the battery is 63.7 °C, and the resistance of the sensor rises from 610 Ω to 860 Ω, implying the temperature is not high enough to activate the PTCR effect. For the 2.5C rate charging at the ambient temperature of 60 °C, the highest internal temperature is detected as 70.2 °C, and the resistance of the sensor increases from 720 Ω to 3030 Ω. An obvious soring of the resistance is observed. During this period, the resistance exceeds 1000 Ω at 67 °C, which is the *T*_c_ of the sensitive element. Therefore, the temperature sensor possesses high resolution to identify the temperature difference in the critical temperature range. Additionally, the time of the *T*_max_ is consistent with that of the current and voltage values in the charging and discharging courses, respectively. It reveals that the sensor recorded the internal temperature variation over time. The performance of the battery with and without a sensor embedded was compared after charging and discharging 500 cycles at 55 °C with a 1C rate (see Figure 10). The results show that the implantation of the sensor caused a decrease in initial capacity of 5% (from 2381 mAh to 2261 mAh). However, the capacity retention of the two batteries during the 500 cycles was almost identical, with 94.8% for the battery with the sensor embedded and 94.3% for the battery without the sensor. This indicates no continued decay in capacitance after the sensor was embedded.

## 4. Conclusions

In summary, a flexible sensor array with BSZYT-BNT sensors was developed. The PTCR sensors show fast and reversible temperature responses. They also have high thermal sensitivity and satisfactory stability. Specifically, the thermal switching temperature of 67 °C matches well with the temperature at which the SEI decomposes. The significant increase in resistance at thermal switching temperatures makes it easy to achieve the switch function. These properties have not been achieved using previous ceramic PTC devices. Batteries embedded with this sensor array show unaffected battery performance at normal temperatures and could be expected to shut down rapidly under abnormal conditions, such as overheating and shorting. They can also resume normal function without compromising performance after intervention and repeated use. Compared with previous approaches, the present work provides a reliable, fast, and reversible strategy that can achieve both unaffected battery performance and improved safety. It could be believable that this strategy holds great promise for practical battery applications.

## Figures and Tables

**Table 1:** Compositions, sintering, and performance parameters of the BSZYT-BNT (0.99(Ba_1-x_Sr_x_Zr_y_Y_0.005_Ti_0.995-y_O_3_)-0.01(Bi_0.5_Na_0.5_TiO_3_)) ceramic samples.

Sample	*ρ*_25_ (Ωcm)	*T*_c_ (°C)	*T*_1_ (°C)	Δ*T* (°C)
*a*	2.3 × 10^5^	70	80.1	−7.6
*b*	8.9 × 10^5^	80	88.6	−20.4
*c*	1.4 × 10^6^	90	94.4	−6.7
*d*	8.1 × 10^3^	67	71.9	−3.3

**Table 2:** Temperature rise rate during thermal runaway experiments.

Test Batteries	Capacity	SOC	Thermal Runaway Triggers	Temperature Rise Rate	
Pouch cell [29]	24 Ah	100%	Thermal abuse	<0.01 °C/min (T<~70 °C) <1 °C/s (~70 °C < T< ~210 °C)	
Cylindrical cell [39]	14,500: 900 mAh; 18,650: 1100 mAh; 26,650: 2500 mAh; 26,650: 3000 mAh	100%	Thermal abuse	≤~1 °C/min (T < 100 °C)	
Prismatic cell [40]	25 Ah	100%	Thermal abuse	≤0.1 °C/min (50 °C < T < 150 °C)	
Pouch cell [41]	7800 mAh	100%	Thermal abuse	<0.02 °C/min (T < ~84.17 °C) <1 °C/min (~84.17–35.88 °C)	
Prismatic cell, Pouch cell [42]	40 Ah (both)	prismatic cell: 148% pouch cell: 154.6%	Electrical abuse	Prismatic cell: ~5.8 °C/min (74–99 °C)	Pouch cell: ~11.2 °C/min (55–93 °C)
Cylindrical cell [17]	3200 mAh	-	Electrical abuse	~1.1 °C/min (~20 °C < T< ~60 °C)	

**Table 3:** The maximum temperature and resistance during the dynamic test.

Environment Temperature	55 °C		60 °C	
Process	2C Charging	2C Discharging	2.5C Charging	2.5C Discharging
*T*_max_ (°C)	63.7	63.3	70.2	69.3
*R*_max_ (×10^3^ Ω)	0.86	0.82	3.03	2.22

## Data Availability

Data are available on request from the authors.
